# First Evidence of Bud Feeding-Induced RNAi in a Crop Pest via Exogenous Application of dsRNA

**DOI:** 10.3390/insects11110769

**Published:** 2020-11-07

**Authors:** Jonathan Willow, Liina Soonvald, Silva Sulg, Riina Kaasik, Ana Isabel Silva, Clauvis Nji Tizi Taning, Olivier Christiaens, Guy Smagghe, Eve Veromann

**Affiliations:** 1 Chair of Plant Health, Institute of Agricultural and Environmental Sciences, Estonian University of Life Sciences, 51006 Tartu, Estonia; liina.soonvald@emu.ee (L.S.); silva.sulg@emu.ee (S.S.); riina.kaasik@emu.ee (R.K.); 2 School of Mental Health and Neuroscience, Faculty of Health, Medicine and Life Sciences, Maastricht University, 6229 ER Maastricht, The Netherlands; silvaai@cardiff.ac.uk; 3 Laboratory of Agrozoology, Department of Plants and Crops, Faculty of Bioscience Engineering, Ghent University, 9000 Ghent, Belgium; tiziclauvis.taningnji@ugent.be (C.N.T.T.); olchrist.christiaens@ugent.be (O.C.); guy.smagghe@ugent.be (G.S.)

**Keywords:** RNA interference, *Meligethes aeneus*, rapeseed, biopesticide, insecticide, Nitidulidae, Coleoptera

## Abstract

**Simple Summary:**

An ecologically sustainable strategy for managing the pollen beetle *Brassicogethes aeneus*, a key pest of oilseed rape (*Brassica napus*) in Europe, is greatly needed. Gene silencing via RNA interference, through sprayed applications of target-specific double-stranded RNA, represents a potential alternative to conventional insecticides. We used dsRNA designed to target a vital gene in this pollen beetle species and allowed the beetles to feed on dsRNA-coated oilseed rape buds. We observed a significant silencing of the target gene; and this was followed by a significant, albeit delayed, reduction in pollen beetle survival rate. Further experiments are necessary in order to better understand the potential for developing a dsRNA-spray approach to pollen beetle management.

**Abstract:**

Spray-induced gene silencing (SIGS) is a potential strategy for agricultural pest management, whereby nucleotide sequence-specific double-stranded RNA (dsRNA) can be sprayed onto a crop; the desired effect being a consumption of dsRNA by the target pest, and subsequent gene silencing-induced mortality. Nucleotide sequence-specificity is the basis for dsRNA’s perceived biosafety. A biosafe approach to pollen beetle (*Brassicogethes aeneus*) management in oilseed rape (*Brassica napus*) agroecosystems is needed. We examined the potential for SIGS in *B. aeneus*, via bud feeding, a field-relevant dsRNA exposure route. Oilseed rape buds were uniformly treated with dsRNA designed to target *αCOP* in *B. aeneus*. Our model control dsRNA (dsGFP) remained detectable on buds throughout the entire 3 d exposure period. When applied at 5 µg/µL, dsαCOP induced significant *αCOP* silencing 3 d after dietary exposure to buds treated with this dsαCOP concentration. We also observed a trend of increased *αCOP* silencing with increasing concentrations of dsαCOP at both 3 and 6 d. Furthermore, we observed a marginally significant and significant reduction in *B. aeneus* survival at 10 and 15 d, respectively. Our results suggest potential for developing a SIGS approach to *B. aeneus* management—though further experiments are needed to more fully understand this potential.

## 1. Introduction

The pollen beetle *Brassicogethes aeneus* Fab. (syn. *Meligethes aeneus*) is a key pest of oilseed rape (*Brassica napus* L.) in Europe. Adult *B. aeneus* overwinter in soil, under vegetation and leaf litter; they emerge in early spring to feed on the pollen and nectar of a variety of blooming plants, and subsequently colonize brassicaceous plants, where they obtain nutrients from reproductive buds and open flowers. After mating, females oviposit into buds, and upon hatching, larvae feed on anthers within buds, eat their way out of the buds, and feed in open flowers, eventually pupating under the soil surrounding the host plant (reviewed in Mauchline et al. [1]). Oilseed rape crops are most susceptible to *B. aeneus* during the green bud stage. Model predictions demonstrate that the extensive bud feeding by *B. aeneus* can result in great economic losses, depending on different factors such as the number of pollen beetles and immigration time [2,3]. Current *B. aeneus* control measures usually occur via the application of synthetic agrochemicals, for example the neonicotinoid insecticide thiacloprid [4,5]. These, however, have shown detrimental effects on nontarget organisms, including hymenopteran parasitoids [6,7,8], a functional group of critical importance for the biocontrol of *B. aeneus* populations [9,10].

To achieve ecologically sustainable oilseed rape production, an integrated and biosafe scheme for *B. aeneus* management is needed. One biosafe approach to *B. aeneus* management is via conservation biocontrol, where habitats and habitat features required by the parasitoids of *B. aeneus* are preserved or restored in oilseed rape agroecosystems, ideally at both local and regional scales [9,10,11,12,13,14]. Insecticide use represents another measure for preventing steep yield losses in oilseed rape production. However, to contribute to a biosafe management design, the insecticidal compounds used must be as specific to the target pest as possible.

Gene silencing via RNA interference (RNAi) represents a potential approach to utilize within integrated pest management [15]. As RNAi occurs via double-stranded RNA’s (dsRNA) nucleotide sequence-specific mode of action, this control measure has potential species-specificity. RNAi efficacy via sprayable dsRNA, known as a spray-induced gene silencing (SIGS) approach, represents a potential strategy for insect pest management in agriculture, the prospects of which are reviewed in Cagliari et al. [16] and Taning et al. [17]; and this approach has indeed been demonstrated, in both a greenhouse experiment [18] and a field trial [19], against the Colorado potato beetle (*Leptinotarsa decemlineata* Say). In contrast to host-induced gene silencing (HIGS) via the use of an RNAi cultivar, a SIGS approach has the benefit of not requiring the biotechnology or time required for engineering an RNAi cultivar.

We recently targeted the vital gene coatomer subunit alpha (*αCOP*), encoding the αCOP protein, and showed RNAi efficacy in *B. aeneus* via honeywater feeding (Willow et al. In Press), indicating potential for RNAi-based control of *B. aeneus* via dsRNA-contaminated nectar. However, *B. aeneus* also requires the lipid and protein constituents of pollen, which they consume from both buds and open flowers. As mentioned above, the most vulnerable stage of oilseed rape growth, with respect to *B. aeneus*, is the green bud stage; as this is the time when *B. aeneus* females oviposit within buds, and both male and female adult *B. aeneus* feed on pollen within buds in order to acquire lipid and protein constituents. Therefore, it is critical to examine RNAi efficacy via bud feeding in *B. aeneus*.

The aim of the present study was to examine RNAi efficacy via a field-relevant and thus far unexamined dietary exposure route, bud feeding, simulating a SIGS approach by uniformly treating bud epithelia. We expected that, by consuming dsRNA-treated bud epithelial tissue, *B. aeneus* individuals would undergo gene silencing and subsequent gene silencing-induced mortality.

## 2. Materials and Methods

A selected 222 bp region from *B. aeneus*’s *αCOP* sequence, and a 455 bp region from the *gene green fluorescent protein* (*gfp*) (Table S1), were the basis for in vitro synthesis of dsRNA by AgroRNA (Genolution, Seoul, South Korea). Both dsRNAs were shipped in distilled water (dH2O) at ambient temperature and kept at 5 ± 1 °C once received. The nucleotide sequences of these dsRNAs were complementary to the genes *gfp* (our control, as *gfp* is not present in insects) and *αCOP* (our target gene). The dsRNAs are hereafter referred to as dsGFP and dsαCOP. The absence of nucleic contaminants in dsRNA products was determined via gel electrophoresis.

Pollen beetles and oilseed rape plants (BBCH 31−32) were both collected from untreated organic oilseed rape fields (beetles: 58.36377°N, 26.66145°E; plants: 58.37389°N, 26.33114°E) in the respective villages of Õssu and Nasja, Tartu County, Estonia. Beetles were kept in ventilated plastic containers, allowed to feed ad libitum on the pollen of oilseed rape flowers, and identified as *B. aeneus* prior to their use in this study. Winter oilseed rape plants were kept in a 3 × 3 m climate room (Flohr Instruments, Utrecht, Netherlands) at 10 °C (70 ± 5% relative humidity and 16:8 h light:dark cycle), in order to maintain them at a low growth stage. Before starting the experiment, the temperature in the climate room was increased to 18 °C. 

Leading racemes, ranging 18‒24 cm in length, were removed from oilseed rape plants during the green bud stage (BBCH 53‒55). Treatments were prepared from dsRNA, dH2O and a constant concentration (180 ppm) of the surfactant Triton X-100 (Fisher Bioreagents) and were vortexed prior to soaking bud clusters. There were three treatments in total, including dsGFP at 5 µg/µL, and dsαCOP at 2.5 and 5 µg/µL. Bud clusters were swirled in treatment solutions for 1 min (this action and duration were both required in order to reliably break the surface tension caused by the waxiness of the bud epithelium), and subsequently allowed to air dry for 1 h. The cut tip of each raceme was then kept underwater, individually, in modified plastic labware (height 12 cm). For each sample, six *B. aeneus* were released within a transparent-white organza fabric bag (20 × 30 cm) that was fastened with string to the neck of the labware. The beetles were allowed to feed ad libitum on the treated buds for 3 d. The 3 d exposure to the dsRNA-treated bud took place in the climate room at 18 °C, 70 ± 5% relative humidity and 16:8 h light:dark cycle.

For each experimental replicate, each treatment was initially allocated five samples; and the experiment was replicated three times. Beetles were not disturbed during their 3 d exposure to dsRNA treatments; thus, survival monitoring began after the 3 d exposure period, and thereafter occurred every 24 ± 1 h. After 3 d of feeding on treated buds, bud-feeding setups were dismantled, and the beetles were transferred to transparent, polystyrene, ventilated insect breeding dishes (diameter 10 cm × height 4 cm) (SPL Life Sciences, Gyeonggi-do, South Korea), hereafter referred to as cages; and the beetles were kept in their respective samples. After this relocation to the laboratory, the beetles were maintained in an incubator (Sanyo MLR-351H, Osaka, Japan) and provisioned daily with fresh untreated oilseed rape flowers, and a dental cotton roll soaked with dH2O. Survival monitoring for each experimental replicate lasted 15 d post-exposure to dsRNA. Escaped beetles were accounted for in the statistical analysis, and any sample where more than two beetles escaped were removed from the analysis at the start of survival monitoring (n = 14 (83 beetles), 14 (80 beetles) 15 (87 beetles), for dsGFP at 5 µg/µL, dsαCOP at 2.5 µg/µL and dsαCOP at 5 µg/µL, respectively). 

Relative gene expression analysis was performed for all treatments via quantitative polymerase chain reaction (qPCR). For each experimental replicate, at 3 d (upon dismantling the bud-feeding setups), and again 6 d after the start of bud feeding, one cage of six live beetles was randomly removed from each treatment (qPCR sample n = 3 per treatment). The removal of beetles for qPCR was accounted for in the statistical analysis. Beetles used for qPCR were immediately placed in their respective Eppendorf tubes and homogenized using a sterilized plastic pestle designed for Eppendorf tubes, in 600 µL of RLT buffer (with added 10 µL of β-mercaptoethanol), and stored at −80 °C until analysis. Total RNA was extracted using an RNeasy Mini Kit (Qiagen, Venlo, Netherlands); and RNA concentration and purity were assessed using a nanodrop spectrophotometer (Thermo Scientific, Wilmington, USA), with purity further verified via gel electrophoresis. Genomic DNA was removed using a Turbo DNA-Free Kit (Invitrogen, Carlsbad, USA), following manufacturer’s instructions. The cDNA was reverse transcribed from 1 µg of total RNA using a FIREScript RT cDNA Synthesis Kit (Solis BioDyne, Tartu, Estonia); and qPCR was performed in the Quantistudio 5 Real-Time PCR System (Applied Biosystems, Foster City, USA). The reaction included 4 µL of 5xHOT FIREPol EvaGreen qPCR Supermix (Solis BioDyne, Tartu, Estonia), 0.5 µL of both 10 µM forward and reverse primers (Microsynth, Balgach, Switzerland; Table S2), 14 µL of PCR-grade water and 500 ng of cDNA, in a total volume of 20 µL. Amplification conditions were 15 min at 95 °C followed by 40 cycles of 15 s at 95 °C and 1 min at 58 °C, and ending with a melting curve analysis with a temperature range of 60‒95 °C. The reactions were set up in 384-well PCR plates, in triplicate. The two housekeeping genes *ribosomal protein S3* (*rps3*) and *actin* (*act*) were used to normalize the data. Primer amplification efficiencies were determined via a cDNA dilution series. Primer sequences and amplification efficiencies are shown in Table S2. Relative gene expression values were calculated using the 2^‒ΔΔCt^ method. A no-template control and a no reverse transcriptase control were included in the assay.

To confirm that dsRNA remained stable over the chosen experimental duration of 3 d, RT-PCR was performed to confirm the presence of dsRNA on buds at the four time points of 1 h, and 1, 2 and 3 d post dsRNA-application. For this, we applied dsGFP at both 2.5 and 5 µg/µL, both treating- and maintaining these bud clusters in the same manner as was performed for bud feeding. At each time point of interest, total RNA was extracted from 0.1 g of buds, using an RNeasy Plant Mini Kit (Qiagen, Venlo, Netherlands), following the manufacturer’s protocol; and RNA concentration was quantified, and purity assessed, using a nanodrop spectrophotometer (Thermo Scientific, Waltham, MA, USA), with purity further verified via gel electrophoresis. The detection of dsGFP was performed from 500 ng of RNA, using a SuperScript III One-Step RT-PCR System (Invitrogen, Carlsbad, CA, USA) with gfp-specific primers at 10 pmol (Table S2). Both 200 ng and undiluted dsGFP were used as positive controls. Samples were run on an Eppendorf Mastercycler (Hamburg, Germany) under the following conditions: 10 min at 75 °C, 30 min at 55 °C, 2 min at 94 °C, 40 cycles of 15 s at 94 °C, 30 s at 55 °C, 1 min at 68 °C, and 5 min at 68 °C. In order to denature the secondary structure of the dsGFP, a denaturing step of 10 min at 75 °C was added to the protocol. The amplified fragments were analyzed via gel electrophoresis.

Regarding both survival- and gene expression analysis, comparisons were made between dsGFP and both concentrations of dsαCOP, and between the two concentrations of dsαCOP. For survival analysis, the homogeneity of variance and normality of data distributions were determined using the Levene and Shapiro–Wilk tests, respectively. Since the data were overall not normally distributed, the Kruskal–Wallis test was used as a nonparametric alternative to ANOVA; this was followed by the Wilcoxon rank-sums test, with Bonferroni correction, for post hoc pairwise comparisons. For gene expression analysis, comparisons were made using Welch’s t-test. All statistical analyses were done in R v3.6.3 (R Foundation for Statistical Computing, Vienna, Austria).

## 3. Results

**Figure 1:**
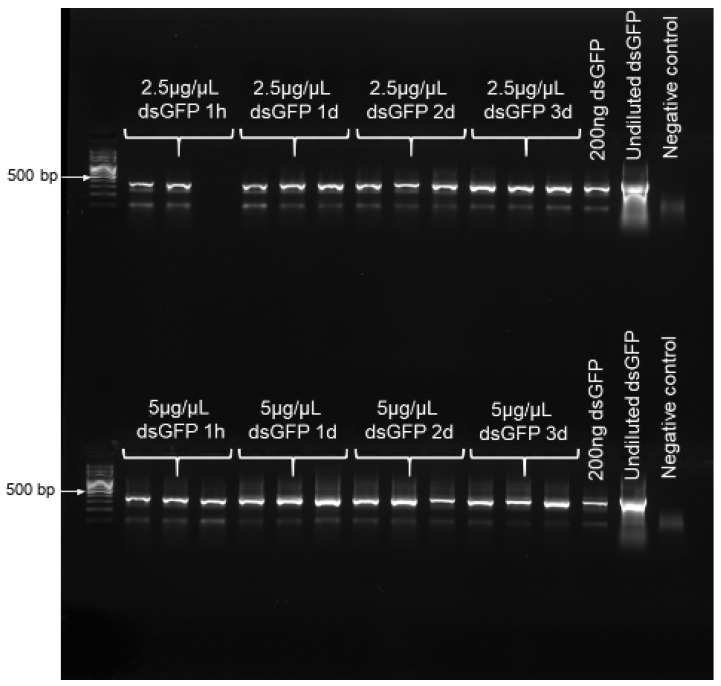
RT-PCR results showing presence of dsRNA (dsGFP applied at both 2.5 and 5 µg/µL) on bud tissue at 1 h, and 1, 2 and 3 d post dsRNA-application.

**Figure 2:**
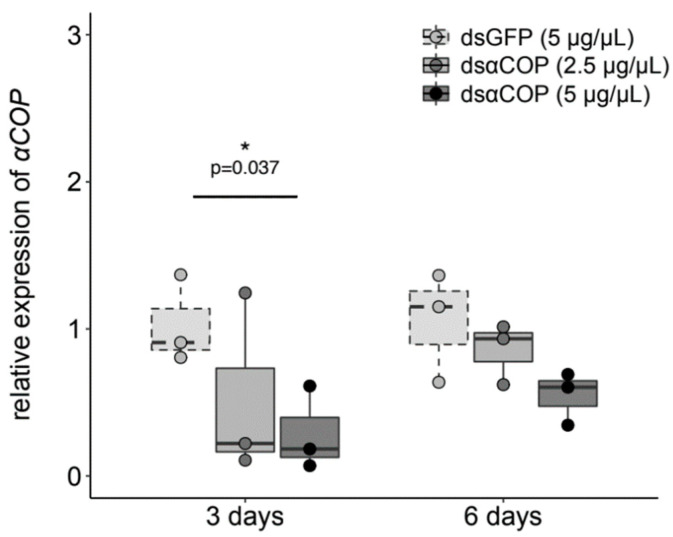
Results of qPCR, showing the relative expression of *αCOP* in *Brassicogethes aeneus* at 3 and 6 d, comparing target treatments (dsαCOP at 2.5 and 5 µg/µL) to dsGFP control. Asterisk (*) indicates significant difference between treatments (analyzed using Welch’s *t*-test).

RT-PCR results confirmed the presence and stability of dsRNA on buds, over the entire 3 d of exposure to treatments, for both dsGFP concentrations examined (Figure 1). In the insects that fed upon the buds, our obtained qPCR results showed a trend of reduced αCOP expression, with an increasing concentration of dsαCOP application, at both 3 and 6 d (Figure 2). At 3 d, we observed a 49% mean decrease in αCOP expression in the dsαCOP 2.5 µg/µL treatment (t = 1.25, df = 2.87, *p* = 0.3), and a 72% mean decrease in the dsαCOP 5 µg/µL treatment (t = 3.09, df = 3.99, *p* = 0.037). At 6 d, we observed a 19% mean decrease in αCOP expression in the dsαCOP 2.5 µg/µL treatment (t = 0.79, df = 3.13, *p* = 0.49), and a 48% mean decrease in the dsαCOP 5 µg/µL treatment (t = 2.11, df = 2.88, *p* = 0.13).

**Figure 3:**
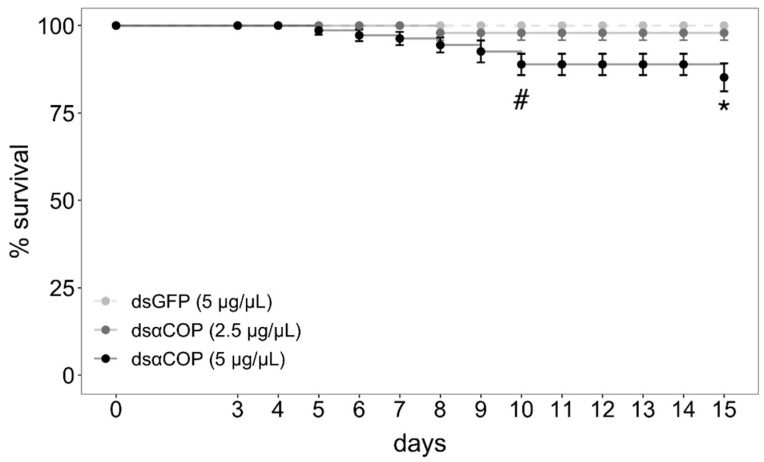
Survival (%) of *Brassicogethes aeneus* in each treatment, accounting for all three experimental replicates. The hash symbol (#) indicates a significant effect of treatment (chi-square). Asterisk (*) indicates a statistically significant difference between dsαCOP treatment and dsGFP control (*p* < 0.05; Kruskal–Wallis test, followed by Wilcoxon rank-sums test with Bonferroni correction).

Regarding survival, we began observing a significant effect of treatment at 10 d (10‒14 d: chi-square = 7.8, df = 2, *p* = 0.02; 15 d: chi-square = 10.38, df = 2, *p* = 0.006; Figure 3). After correcting for pairwise comparisons, mortality in the dsαCOP 5 µg/µL treatment was marginally significant at 10‒14 d (*p* = 0.056), becoming significant at 15 d (*p* = 0.021). Survival in this treatment slowly fell from 100% (4 d) to 99 (5 d), 97 (6 d), 96 (7 d), 94 (8 d), 92 (9 d), 88 (10 d) and 84% (15 d). No significant effect on survival was observed for the dsαCOP 2.5 µg/µL treatment, survival falling from 100% (7 d) to 98% (8 d), where it settled. No mortality was observed in the dsGFP treatment. After the 3 d treatment–exposure period, all bud clusters had numerous buds incised, with both anthers and bud epithelium consumed. Together with the fact that all caged beetles survived over the entire 3 d treatment–exposure period, which indicates that all beetles fed on dsRNA-treated bud tissue.

## 4. Discussion

We provide laboratory evidence suggesting some potential for incorporating a SIGS approach within integrated *B. aeneus* management. We observed marginally significant and significant reductions in survival at 10 and 15 d, respectively, as well as a trend of lower relative expression of *αCOP* with increasing concentrations of dsαCOP, indicating that the mortality observed in our *B. aeneus* RNAi assays were a result of silencing the target gene *αCOP*. However, while we suggest some potential for SIGS in *B. aeneus* management via dsRNA-treated buds, there is certainly more to be explored here before this idea can be further developed. 

We treated the oilseed rape bud epithelia, where *B. aeneus* chews through and consumes this tissue mostly to obtain nutrients from the anthers within. If *B. aeneus*-specific dsRNA formulations were to exhibit properties that allow the dsRNA to absorb past the bud epithelium, and into the anthers within, a SIGS approach utilizing such formulations would likely show greater RNAi efficacy. Furthermore, as *B. aeneus* development begins within the reproductive bud, and larvae are in their late first- or early second instar when oilseed rape buds blossom, it is plausible that such an approach could target both larval and adult *B. aeneus* simultaneously. Studies examining the potential for RNAi in *B. aeneus* larvae, via the use of co-formulants to enhance the transport of dsRNA past the bud epithelium, would be of great value to our understanding of the potential for *B. aeneus* management via SIGS. Moreover, as adults of *B. aeneus* appear to show modest RNAi-sensitivity, it would be of great value to investigate whether *B. aeneus* larvae are more RNAi-sensitive than adults, as this would further guide research endeavors to target this larval life-stage of this species.

With regard to adult bud feeding, it is possible that a duration of dsRNA exposure greater than 3 d is necessary for inducing RNAi at a quicker rate and in a higher percent of the sample. While a longer exposure duration is likely to be especially crucial, this would be limited by the duration of oilseed rape’s bud stage, as well as the total length of time that the applied dsRNA-based insecticide remains present and stable on- and in the oilseed rape bud under field conditions. Both the duration of bud stage and the duration of dsRNA stability will undoubtedly vary depending on environmental conditions. However, the results of a small-scale field trial near Ljubljana, Slovenia, using sprayed naked dsRNA for the control of *L. decemlineata*, showed that the sprayed dsRNA remained stable long enough to have the desired effect under natural environmental conditions [19]. As oilseed rape’s flowering structures (i.e., reproductive buds, bloomed flowers) are in constant development and senescence, the possibility of requiring successive dsRNA spray applications must be considered.

While RNAi will likely never result in target pest mortality as quickly as seen in some other (e.g., neurotoxic) insecticides, there are great benefits to using dsRNA-based insecticides due to the associated biosafety to nontarget organisms, stemming from its unique mode of action. Moreover, there remains potential for increasing speed-to-effect via co-formulants (e.g., nanoparticles) that may improve dsRNA-uptake and RNAi efficiency [20,21]. Improving the efficacy of this technology, with regard to *B. aeneus* control via bud feeding, will be a critical aspect to explore if we are to more fully realize the potential for using a SIGS approach in *B. aeneus* management.

## 5. Conclusions

Ecologically sustainable control measures are greatly needed in oilseed rape production; and dsRNA-based insecticides, due to their mode of action, represent a potentially species-specific complement to other biosafe measures (e.g., conservation biocontrol) for managing *B. aeneus*. While our work suggests potential for developing a SIGS approach for implementation in *B. aeneus* management, further experiments are needed to more fully explore the potential for incorporating this approach. Focal points necessary for progress here include determining the potential for enhancing adult *B. aeneus* control efficacy, and that of both larval and adult *B. aeneus* simultaneously, via the use of co-formulants to enhance the transport of dsRNA to anthers within oilseed rape buds. Other important focus points include determining the total duration at which exogenously-applied dsRNA remains viable both on- and within the reproductive bud; and determining the optimal duration of exposure to dsRNA-treated buds, taking into account the time-to-flowering of buds. Finally, it will be critical to determine the overall feasibility of using a SIGS approach in the context of a potential requirement for successive dsRNA spray applications.

## Supplementary Materials

The following are available online at https://www.mdpi.com/2075-4450/11/11/769/s1, Table S1: *Green fluorescent protein* (*gfp*) and *Brassicogethes aeneus coatomer subunit alpha* (*αCOP*) regions for in vitro dsRNA synthesis. Table S2: Primer amplification efficiencies of genes used in the present study.
