# Supplementary material for: First Evidence of Bud Feeding-Induced RNAi in a Crop Pest via Exogenous Application of dsRNA
Source: Insects. 2020 Nov 7;11(11):769. doi: 10.3390/insects11110769 (PMC7694955; doi:10.3390/insects11110769)
Supplement: Supplementary file 1 [file insects-11-00769-s001.pdf]

Supplementary material

Table S1. *Green fluorescent protein* (*gfp*) and *Brassicogethes aeneus coatomer subunit alpha* (*αCOP*) regions for *in vitro* dsRNA synthesis.

Gene	Name	Length of region (bp) for dsRNA synthesis	Sequence of gene region
αCOP	*coatomer subunit alpha*	222	AAGATGCACCACATAATGGACATTTTTCTTCAG GTTTTCCTCTGTAAATGGGGGTGTATGAATAAC CACAAATCGAAAATGGATTATGTTCATCGTAAT TTAATTGAAGTTCATCTGTTAAATTAGAGTCAC AGGCCTGTAAAATTTTTCTTGCCTGCTGAGCCA CCTCAGGACGTGGGCCCAATTCCAATAGCCTCT TGGCCAGCGAAGACG
gfp	*green fluorescent protein*	455	TACGGCGTGCAGTGCTTCAGCCGCTACCCCGAC CACATGAAGCAGCACGACTTCTTCAAGTCCGCC ATGCCCGAAGGCTACGTCCAGGAGCGCACCATC TTCTTCAAGGACGACGGCAACTACAAGACCCGC GCCGAGGTGAAGTTCGAGGGCGACACCCTGGT GAACCGCATCGAGCTGAAGGGCATCGACTTCA AGGAGGACGGCAACATCCTGGGGCACAAGCTG GAGTACAACTACAACAGCCACAACGTCTATATC ATGGCCGACAAGCAGAAGAACGGCATCAAGGT GAACTTCAAGATCCGCCACAACATCGAGGACG GCAGCGTGCAGCTCGCCGACCACTACCAGCAG AACACCCCCATCGGCGACGGCCCCGTGCTGCTG CCCGACAACCACTACCTGAGCACCCAGTCCGCC CTGAGCAAAGACCCCAACGAGAAGCGCGATCA

Table S2. Primer amplification efficiencies of genes used in the present study.

Gene	Name	Forward (5' to 3')	Reverse (5' to 3')	PCR type and efficiency (%)
gfp	*green fluorescent protein*	CACATGAAGCAGCACGACTT	TGCTCAGGTAGTGGTTGTCG	RT-PCR
αCOP	*coatomer subunit alpha*	CCAACTTGGTCATTTAACAATCTGG	CCCTTCCTTCATCATGGACA	qPCR, 107.8
rps3	*ribosomal protein S3*	CCAACGCGTACCGAAATTAT	GAGTTTTCGGGGAAGTTGAA	qPCR, 100
act	*actin*	TCACGGACAATTTCCCTTTC	TATCCTCCGTTTGGACTTGG	qPCR, 90.8
